# Prolonged zinc use for dysgeusia causing copper deficiency

**DOI:** 10.1017/S1478951525101478

**Published:** 2026-01-16

**Authors:** Min Ji Kim, Michael Jay Tang, Eduardo Bruera

**Affiliations:** Department of Palliative Care, Rehabilitation, and Integrative Medicine, The University of Texas MD Anderson Cancer Center, Houston, TX, USA

**Keywords:** Zinc, dysgeusia, copper deficiency, hypocupremia, zinc supplementation

## Abstract

**Background:**

Zinc is a micronutrient essential for taste perception and may be prescribed for dysgeusia in cancer patients undergoing treatment. However, overconsumption of zinc can lead to copper deficiency, which is likely under-recognized and can present as fatigue, nausea, anemia, and myelopathy.

**Case presentation:**

A patient in his 70s with multiple myeloma and gastroparesis taking zinc supplementation to treat dysgeusia for the past 2 years presented with generalized fatigue, lightheadedness, nausea, neutropenia, anemia, gait disturbance, and worsening numbness and tingling in the bilateral lower extremities and hands. He was found to have hypocupremia in the setting of prolonged zinc supplementation and admitted for inpatient treatment with IV cupric chloride. His symptoms gradually improved over the course of approximately 5–6 weeks.

**Conclusions:**

Clinicians should be vigilant about screening for copper deficiency symptoms in patients taking zinc supplementation and avoid prolonged courses or overprescribing of zinc. Hypocupremia should be promptly diagnosed and treated to prevent permanent neurological deficits.

## Introduction

Taste distortion or dysgeusia is a common symptom among cancer patients, with prevalence ranging from 22% to 85% of cancer patients receiving chemotherapy (Di Meglio et al. 2022; Ito et al. 2022). Chemotherapy may have neurotoxic effects or inhibit the differentiation or proliferation of taste buds, impacting taste and smell (Henkin 1994). Sustained inflammatory state, disruptions in the gut microbiota, radiation to the head and neck causing damage to taste cells, xerostomia, and oral mucositis may be other causes (Murtaza et al. 2017). Dysgeusia can have many negative downstream effects such as decreased appetite, malnutrition, weight loss, cachexia, and worsened quality of life (Holmes 1993; Rosati et al. 2024; Suka et al. 2024; Liang et al. 2025).

Zinc is a micronutrient essential for taste perception. Some studies have associated zinc deficiency with dysgeusia in cancer patients (Heyneman 1996; Yamagata et al. 2003), with cancer treatment drugs theorized to affect the binding and chelation of zinc (Comeau et al. 2001). Daily zinc supplementation ranging from 25 to 100 mg have been suggested to treat dysgeusia for patients who may have zinc deficiency (Heyneman 1996).

The effects of zinc overconsumption should be considered, however, for clinicians who are inclined to prescribe zinc empirically for dysgeusia in cancer patients. Short-term effects include gastrointestinal symptoms such as nausea, vomiting, abdominal pain, diarrhea, as well as fatigue, weakness, and dizziness (Fosmire 1990). Long-term effects include copper deficiency, anemia and neutropenia, impaired immune function, hepatic dysfunction, and neuronal damage (Lemire et al. 2008; Morris and Levenson 2017). This case report describes a cancer patient receiving prolonged zinc supplementation who developed worsening symptoms and was later found to have copper deficiency requiring immediate treatment.

## Case presentation

### Initial presentation

A patient in his 70s with multiple myeloma being treated with chemotherapy and prior auto-stem cell transplant was seen in the hematology clinic for evaluation of neutropenia. Other past medical history included gastroesophageal reflux disease, Barrett’s esophagus, gastroparesis, and benign prostatic hyperplasia (BPH). White blood cell count at the time of hematology referral was 1.5 K/µL (neutrophil 29.2%), not fully attributable to his multiple myeloma or treatment-related effects, and hemoglobin ranging from 7.7 to 8.6 g/dL. As for symptoms, he reported progressive fatigue, nausea, and poor appetite for the past few weeks. He also stated having worsening numbness and tingling pain extending from the bilateral lower extremities to the thighs and new onset tingling in the bilateral hands for the past few weeks, in the background of known chemotherapy-induced peripheral neuropathy. His home medications included acyclovir 800 mg twice a day and sulfamethoxazole-trimethoprim 800 mg-160 mg three times a week for infection prophylaxis, amoxicillin-clavulanate 875 mg-125 mg twice a day following a recent hospitalization for fever, morphine sulfate immediate release 7.5 mg every 4 h as needed for cancer-related pain, alfuzosin 10 mg daily and finasteride 5 mg daily for BPH, and zinc sulfate 220 mg twice a day for dysgeusia initially prescribed approximately 2 years ago.

For the neutropenia, lab tests to investigate autoimmune etiologies (rheumatoid factor [RF], anti-neutrophil antibody [ANA]) and a copper level were ordered. The patient’s ANA and RF factors were undetectable. However, he was found to have a very low copper level of < 10 mcg/dL. Due to worsening lightheadedness, nausea, fatigue, and the finding of hypocupremia, he was instructed to present to the acute care center.

### Hospital course

The patient was admitted to the hospital for further management. Zinc was discontinued. IV cupric chloride infusion of 4 mg daily was initiated with the plan to administer for a total of 10 days.

The supportive and palliative care team was consulted to assist with symptom management. The patient reported painful neuropathy with tingling and numbness in the bilateral lower extremities extending to the upper thighs and bilateral hands. He declined gabapentin or duloxetine; gabapentin had been ineffective, and he had discontinued duloxetine due to side effect concerns. He also reported increase in his chronic lower back pain related to vertebral compression fractures and lumbar facet arthropathy. Morphine immediate release oral solution 5 mg every 4 h as needed was prescribed to treat lower back pain and severe pain from neuropathy. Abdominal imaging revealed constipation, which was treated with senna tablets and polyethylene glycol daily. He reported that dysgeusia was no longer an issue.

For nausea with history of gastroparesis, gastroesophageal reflux disease, and Barrett’s esophagus, the gastrointestinal medicine team was consulted. It was determined that nausea was likely multifactorial due to severe copper deficiency exacerbating the known gastroparesis. He was given erythromycin three times a day for 3 days.

The neurology team was also consulted to assess the peripheral neuropathy. On further neurologic exam, he was found to have impaired gait, decreased vibration sense in the anterior superior iliac spine and ankles bilaterally, as well as a positive Romberg test. The findings were concerning for subacute combined degeneration of the spinal cord related to copper deficiency. Additional lab work to check a vitamin B12 level found this to be within normal range at 945 pg/mL. The neurology team recommended further copper repletion.

### Outcome

The patient reported improvement in fatigue, nausea, and pain by hospital day 3, as seen in his Edmonton Symptom Assessment System (ESAS) scores (Table 1). He also reported mild improvement in paresthesia of the lower extremities during his hospital course. He received 5 days of IV cupric chloride inpatient. He was discharged with the plan to continue additional copper infusions in the outpatient setting for 5 more days.

**Table 1.** Edmonton Symptom Assessment System (ESAS) scores

Symptom	Hospital day 2	Hospital day 3	Post-discharge day 39
Pain	8	4	5
Fatigue	10	5	7
Nausea	10	0	5
Depression	0	0	0
Anxiety	0	0	0
Drowsiness	6	0	5
Shortness of breath	0	0	0
Appetite	3	5	4
Well being	10		4
Sleep problems	6	2	2
Total score	63	16	32

He was seen in the supportive care clinic 39 days post-discharge and reported some symptom improvement. Fatigue and pain remained improved overall from his hospital admission. He was not using any medications for pain or neuropathy. He reported the numbness and tingling in his lower extremities had diminished to the knee level. He denied dysgeusia but did report persistent issues with early satiety, nausea, and constipation. He was advised to increase laxatives and take metoclopramide 5 mg three times daily for nausea and early satiety.

## Discussion

Copper is an essential mineral for cellular processes including energy production, red blood cell formation, neurological, and immune system functions (Scheiber et al. 2013). Copper deficiency caused by zinc supplementation has been described in prior literature (Gupta and Carmichael 2023; Magham et al. 2023). Both copper and zinc are absorbed in the gastrointestinal tract, primarily in the stomach and small intestine. Excess zinc increases copper-binding protein metallothionein in the enterocytes, causing copper to be bound and eliminated with sloughing of intestinal cells; this ultimately leads to copper depletion (Halfdanarson et al. 2008).

The evidence for zinc supplementation to treat dysgeusia in cancer patients has been mixed. Some studies have found zinc can improve dysgeusia, especially in head and neck cancer patients (Silverman and Thompson 1984; Ripamonti et al. 1998; Najafizade et al. 2013), while others have shown no benefit (Halyard et al. 2007; Lyckholm et al. 2012; Khan et al. 2019). Polaprezinc, a chelated compound combining zinc and L-carnosine, was found to improve dysgeusia in pancreatic cancer (Fujii et al. 2018) and head and neck cancer patients (Watanabe et al. 2010), while no significant difference in xerostomia or taste was found for patients with hematologic malignancy (Hayashi et al. 2014).

Clinicians should be aware of additional limitations to zinc supplementation for dysgeusia. First, it is challenging to detect a deficiency in zinc, as serum zinc concentration is not a reliable indicator of insufficiency (King 1990). Therefore, zinc supplementation is usually initiated on an empiric basis. Second, the serum zinc level has not been found to be associated with taste acuity (Bales et al. 1986), and it is difficult to determine when zinc has been adequately supplemented. Therefore, it may be prudent for clinicians to prescribe zinc supplementation with a predetermined endpoint in mind, such as 2 months of total treatment to reduce the risk for copper deficiency.

Non-pharmacologic strategies for dysgeusia have a low risk to benefit ratio for cancer patients and should also be discussed. For example, oral care with chlorohexidine and sodium bicarbonate mouthwashes can help with dysgeusia (Magnani et al. 2019). Nutritional counseling, flavor enhancement, and chemosensory education may also improve nutritional intake and taste perception (Schiffman et al. 2007; Rehwaldt et al. 2009). Clinicians may also provide counseling that some taste and smell alterations can recover months after completion of cancer treatment (de Vries et al. 2018).

Copper deficiency may be easily overlooked, especially in cancer patients who often have high symptom burden due to multiple factors such as chemotherapy or the cancer itself. It is an under-recognized cause for cytopenias and myelopathy that is similar in presentation and imaging as vitamin B12 deficiency (Kumar 2006). Timely recognition and treatment with copper repletion are critical; while cytopenia may be reversible with treatment, neurological deficits to the spinal cord and peripheral nerves may become permanent (Gabreyes et al. 2013). This case further highlights the importance of recognizing zinc supplementation as a cause for copper deficiency. Clinicians should screen for signs of hypocupremia in patients who are taking zinc for dysgeusia and be mindful of overprescribing or prolonged use.
